# Spatial modeling of data with excessive zeros applied to reindeer pellet‐group counts

**DOI:** 10.1002/ece3.2449

**Published:** 2016-09-12

**Authors:** Youngjo Lee, Md. Moudud Alam, Maengseok Noh, Lars Rönnegård, Anna Skarin

**Affiliations:** ^1^ Department of Statistics Seoul National University Seoul Korea; ^2^ School of Technology and Business Studies Dalarna University Falun Sweden; ^3^ Department of Statistics Pukyong National University Busan Korea; ^4^ Department of Animal Nutrition and Management Swedish University of Agricultural Sciences Uppsala Sweden

**Keywords:** excessive zeros, habitat preference, hierarchical generalized linear model, pellet‐group count, Poisson model, spatial correlation

## Abstract

We analyze a real data set pertaining to reindeer fecal pellet‐group counts obtained from a survey conducted in a forest area in northern Sweden. In the data set, over 70% of counts are zeros, and there is high spatial correlation. We use conditionally autoregressive random effects for modeling of spatial correlation in a Poisson generalized linear mixed model (GLMM), quasi‐Poisson hierarchical generalized linear model (HGLM), zero‐inflated Poisson (ZIP), and hurdle models. The quasi‐Poisson HGLM allows for both under‐ and overdispersion with excessive zeros, while the ZIP and hurdle models allow only for overdispersion. In analyzing the real data set, we see that the quasi‐Poisson HGLMs can perform better than the other commonly used models, for example, ordinary Poisson HGLMs, spatial ZIP, and spatial hurdle models, and that the underdispersed Poisson HGLMs with spatial correlation fit the reindeer data best. We develop R codes for fitting these models using a unified algorithm for the HGLMs. Spatial count response with an extremely high proportion of zeros, and underdispersion can be successfully modeled using the quasi‐Poisson HGLM with spatial random effects.

## 1 Introduction

Fecal pellet‐group counts have long been used in wildlife management to map population densities of large herbivores and their habitat selection (see, e.g., Fattorini, Ferretti, Pisani, & Sforzi, 2011; Neff, 1968; Skarin, 2008). The technique provides managers with a simple and cheap alternative to modern technologies, such as GPS collars for the surveillance of animal populations (Edge & Marcum, 1989). Although the pellet‐group counts provide only a crude indication of animal habitat use rather than more precise measures of movement and habitat selection which, for example, GPS tracking can do, they still give a general idea of species distributions over different geographic areas and information about habitat use by all animals in a population using a defined area. However, an awareness of pellet decay is necessary in order to interpret the data correctly (e.g., Davis & Coulson, 2016; Skarin, 2008).

A reindeer pellet‐group survey was conducted in the northern forest area of Sweden in order to assess the impact of newly established wind farms on reindeer habitat selection. From the initial survey data, collected over the 2 years 2009–2010, appearance of large numbers of 0 counts was identified as a challenge for the data analysis. This situation is not unusual in that data pertaining to spatial species counts often contain excessive zeros (see, e.g., Agarwal, Gelfand, & Citron‐Pousty, 2002; Dénes, Silveira, & Beissinger, 2015; Zuur, Saveliev, & Ieno, 2012), and requires appropriate modeling.

In the literature, zero‐inflated Poisson (ZIP; Lambert, 1992), hurdle models (Cragg, 1971), and their extensions are widely suggested for modeling counts with excessive zeros (Zuur et al., 2012). Recently, Neelon, Ghosh, and Loebs (2013) used a spatial (Poisson) hurdle model to analyze hospital emergency department visits. Agarwal et al. (2002) used a ZIP model for modeling spatial species counts. Ver Hoef and Jansen (2007) used a spatiotemporal ZIP model for analyzing data on Harbor seal counts. Agarwal et al. (2002) and Neelon et al. (2013) used intrinsic conditional autoregressive (ICAR) structure, whereas Ver Hoef and Jansen (2007) used conditional autoregressive (CAR) random effects to handle spatial correlation, and all of them took a Bayesian approach to their model computations, using Markov Chain Monte Carlo (MCMC) simulations.

Even though the hurdle and ZIP models are often found to be suitable for analyzing data with excessive zero counts, these models apply only when there is overdispersion in the response variable. However, in many applications it has been found that excessive zero counts are associated with underdispersion (see, e.g., Oh, Washington, & Nam, 2006; Tin, 2008) for which ZIP and hurdle models do not make sense. Ridout and Besbeas (2004) presented two examples closely related to ecology, one pertaining to variability in birds’ clutch size and another on polyspermy of eggs, which showed underdispersion. Unfortunately, any discussion of the issue of underdispersion associated with excessive counts has been missing from ecological applications. One possible reason might be that the Bayesian computational software (e.g., WinBugs; Lunn, Thomas, Best, & Spiegelhalter, 2000) restricts researchers to including only overdispersion with zero‐inflated count responses. Theoretically, given that the mean is correctly specified as a function of the covariates, underdispersion can appear with count data if the underlying data are generated, for instance, from a double‐Poisson (Efron, 1986), weighted Poisson (Ridout & Besbeas, 2004), Poisson mixture (e.g., generalized Poisson; Consul & Jain, 1973), or gamma (Oh et al., 2006) distribution. A failure to account for the correct type of over‐/underdispersion with count data can lead to the model having a poor fit of the model (Ridout & Besbeas, 2004; Tin, 2008), very different estimates of the regression parameters (Ver Hoef & Boveng, 2007), and incorrect inferences about the model parameters (McCullagh & Nelder, 1989).

In this study, we show that high spatial correlation among counts can result in excessive zeros. Thus, to fit the reindeer pellet‐group counts, we investigate whether an over‐/underdispersed quasi‐Poisson hierarchical generalized linear model (HGLM; Lee, Nelder, & Pawitan, 2006) with spatial correlation is suitable. The difference between a Poisson model, *y* ~ Poisson(λ), and a quasi‐Poisson model is that the variance of *y* is λ for a Poisson model, whereas the variance of *y* in a quasi‐Poisson model includes an additional parameter ϕ such that var(*y*) = ϕλ (Nelder & Pregibon, 1987). For ϕ > 1 (or ϕ < 1), the data are referred to as overdispersed (or underdispersed).

While a spatial hurdle model only allows for overdispersion, a spatial quasi‐Poisson HGLM allows for either over‐ or underdispersion. The HGLM approach can confer computational advantages because the spatial quasi‐Poisson HGLMs and the spatial hurdle model can both be fitted using the iterative weighted least square (IWLS) algorithm developed by Lee and Nelder (1996) for HGLMs. Furthermore, the HGLM approach enables us to compare these alternatives, via conditional Akaike's information criterion (cAIC; Lee et al., 2006) and adjusted profile likelihoods. Lee and Nelder (1996) extended the scaled deviance and its degrees of freedom for GLMs to HGLMs. Based on these, the deviance information criterion (DIC) has been proposed as a model selection criterion (Spiegelhalter, Best, Carlin, & van der Linde, 2002), while cAIC was developed as a model selection criterion in frequentist work (Vaida & Blanchard, 2005). For comparison of these two information criteria (see Section 6.5 of Lee et al., 2006).

The aim of this study is to show how a spatially correlated count response with excessive zeros can be successfully modeled using HGLMs. We achieve this by: (1) presenting the HGLMs for spatially correlated count data; (2) providing a theoretical comparison of the HGLMs with zero‐inflated count data models; and (3) applying HGLM to a real data set pertaining to reindeer pellet‐group counts and comparing fits of HGLMs with those of the spatial hurdle and zero‐inflated models. Brief descriptions of the estimation techniques and their R (R Core Team, 2014) implementations are provided in the supplementary material. Here, it should be noted that the HGLM methodology, using IWLS, can fit the following models that include spatial correlation: Poisson generalized linear mixed model (GLMM), quasi‐Poisson HGLM, and hurdle model. We use the reindeer pellet‐group counts as an example showing the need for a quasi‐Poisson model with spatially correlated random effects in ecological modeling. It is the only model of those investigated in this study that can accommodate both underdispersion and excess zeros.

## 2 Materials and Methods

Lee and Nelder (1996) presented HGLMs to model correlated exponential family responses by incorporating independent random effects. These models were extended to deal with correlated random effects in Lee et al. (2006). In the following, we briefly introduce a Poisson HGLM with normally distributed random effects, that is, a GLMM. Thereafter, we show how a quasi‐Poisson HGLM with correlated random effects provides a way to model excess zeros jointly with over‐/underdispersion. For comparison, we also present two models commonly used for data with excess zeros: the hurdle model and the zero‐inflated negative‐binomial model. For the different models, we also present a theory explaining how they can fit over‐ or underdispersion. At the end of the section, we present the example data set for reindeer pellet‐group counts.

### 2.1 Poisson GLMM

Consider a Poisson GLMM of counts *z*_*i*_ or *i *=* *1,2,…,*n* with conditional mean λ_*i*_ = E(*z*_*i*_|*v*_*i*_)

(1) $$z_{i}|v_{i}∼\text{Poisson}\left(\lambda_{i}\right),$$

(2) $$\eta_{i}=X_{i}\beta +v_{i},$$

(3) $$v_{i}∼N\left(0,\tau \right),$$

where *X*_*i*_ is a row vector of covariates, and β and τ are fixed parameters.

In this model,

$$\mu_{i}=E\left(z_{i}\right)=E\left(E\left(z_{i}|v_{i}\right)\right)=\exp\left[X_{i}\beta +\frac{1}{2}\tau \right],$$

and

$$\text{Var}\left(z_{i}\right)=E\left(\text{Var}\left(z_{i}|v_{i}\right)\right)+\text{Var}\left(E\left(z_{i}|v_{i}\right)\right)=\mu_{i}+a\mu_{i}^{2},$$

where a=exp[τ]−1≥0. Clearly, $\text{Var}\left(z_{i}\right)\geq \mu_{i}$ for τ ≥ 0, where the equality holds for τ = 0. Thus, the GLMM automatically accounts for overdispersion, which resulting in more zero counts than a Poisson GLM (this issue is further elaborated in Section 3.2).

### 2.2 Quasi‐Poisson HGLM with spatial correlation

The spatial latent intensity approach for spatial count data was presented by Clayton and Kaldor (1987) and was subsequently modified by many others, including Cressie (1993) and Lee et al. (2006). The basic model is as follows. Given a random intensity λ_*i*_ for location *i* (*i* = 1,2,…,*n*), which is identified by the spatial coordinates *s*(*i*) = (*x*_*i*_, *y*_*i*_), the conditional (count) response process *z*_*i*_ follows a double exponential family (Lee et al., 2006; equivalent to the extended quasi‐Poisson model, Efron, 1986), that is,

(4) $$f\left(z_{i}|v_{i}\right)=\phi^{-\frac{1}{2}}\exp\left[-\frac{\lambda_{i}}{\phi }\right]\frac{\exp\left[\left(\frac{1}{\phi }-1\right)z_{i}\right]z_{i}^{z_{i}}}{z_{i}!}{\left(\frac{\lambda_{i}}{z_{i}}\right)}^{z_{i}/\phi },$$

(5) $$\approx \phi^{-1}\exp\left[-\frac{\lambda_{i}}{\phi }\right]\frac{{\left(\lambda_{i}/\phi \right)}^{z_{i}/\phi }}{\left(z_{i}/\phi \right)!},$$

where ϕ is the dispersion parameter. This model gives *E*(*z*_*i*_|*v*_*i*_) = *λ*_*i*_ and $\text{Var}\left(z_{i}|v_{i}\right)=\phi \lambda_{i}$; ϕ = 1 gives the Poisson distribution. It allows for overdispersion when ϕ > 1 and underdispersion when ϕ < 1. Equation (5) can be obtained from equation (4) using Stirling's approximation and this was used to formulate the extended quasi‐likelihood by Nelder and Pregibon (1987). Lee and Nelder (2000) showed that equations (4) and (5) give identical likelihood inferences.

Lee et al. (2006, Section 7.2) showed that the use of a quasi‐Poisson‐GLM can give inefficient estimate for dispersion parameter ϕ when the data are generated from a random‐effect model. Zuur et al. (2012) also showed, in a simulation study, that quasi‐Poisson‐GLM should not be used to model overdispersion due to zero inflation. Lee et al. (2006) proposed that instead of quasi‐Possion‐GLM, the use of quasi‐Poisson‐HGLM allowing for an additional random effect *v*_*i*_ produces a better fit. Thus, in this study, we propose the use of quasi‐Possion‐HGLM for count data with excessive zeros.

Further, we model the random intensity parameter λ_*i*_ as

(6) $$\log\left(\lambda_{i}\right)=X_{i}\beta +v_{i},$$

where *v*_*i*_ is a random location effect following a certain distribution. It is generally assumed that **v** ^T^ = (*v*_1,_ *v*_2,_ … *v*_*n*_) follows a multivariate normal distribution, that is, **v** ~ *N*(0, Σ).

One popular structure of Σ for spatial covariance is $\Sigma =\tau {\left(I-\rho D\right)}^{-1}$ where **I** is an identity matrix, **D** is a known symmetric matrix and Σ is positive definite giving the so called CAR structure (Besag, 1974) for **v**. However, the construction of the **D** matrix needs some careful consideration, which has been discussed elsewhere (see, e.g., Cressie, 1993; Haining, 1990; Wall, 2004).

Besides CAR (proper), other popular choices for the joint distribution of **v** includes the intrinsic CAR (or ICAR; Besag & Coperberg, 1995; Neelon et al., 2013; Lee et al., 2006) and the spatial (or simultaneous) autoregression (Ord, 1975; Wall, 2004) which gives **v**~*N*(0, ((**I** − *ρ* **D**)^T^(**I** − *ρ* **D**))^−1^). All these correlation structures, CAR, ICAR, and SAR, can be fitted using a HGLM fitting algorithm (R implementations are provided in the supplementary materials).

For a CAR model (as in Section 2.1), following Lee et al. (2006) and Ver Hoef and Jansen (2007), we constructed **D** = {*d*_*i*,*j*_} as

$$d_{i,j}=\left\{\begin{array}{cc} \frac{1}{\left||s(i)-s(j)|\right|} & \text{if}\;i\neq j\\ 0 & \text{if}\;i=j \end{array},\right.$$

where ||*s*(*i*) − *s*(*j*)|| represents the Euclidean distance between locations *i* and *j*.

### 2.3 Hurdle and ZIP models for spatial zero‐inflated counts

In the literature, zero‐inflated Poisson (ZIP) and hurdle models, and their extensions are widely suggested for modeling counts with excessive zeros. The arguments in favor of ZIP and hurdle models, besides any background theory about the actual data generation process, are as follows. First, zero‐inflated data contain more zeros than can be generated by an ordinary Poisson model for count data. Second, in the presence of zero inflation, the mean–variance relationship within an ordinary count data model breaks down. And third, the zero‐inflated (and mixture) model can handle possible multiple modes (one mode at zero) in the data.

A hurdle model can be presented as follows. Given $v_{0}={\left\{v_{0,i}\right\}}_{i=1}^{n}$ and $v_{1}={\left\{v_{1,i}\right\}}_{i=1}^{n}$, the response follows

(7) $$\left.\begin{array}{ccc} \mathrm{Pr}(y_{i}=y|v_{0,i},v_{1,i},X_{i}) & =\mu_{0,i} & \text{if}\;y=0\\ & =\left(1-\mu_{0,i}\right)\text{TP}\left(y,\mu_{1,i}\right) & \text{if}\;y=1,2,\ldots \\ g_{k}\left(\mu_{k,i}\right) & =X_{k,i}\beta_{k}+Z_{ki}v_{k} & k=0,1 \end{array},\right\},$$

where TP is a zero‐truncated Poisson probability mass function, *g*_*k*_ is “logit” and “log” link for *k *=* *0 and 1, respectively, and **v**_*k*_ values are assumed to follow some sort of multivariate distribution (e.g., Gaussian CAR or SAR), which account for the spatial correlation. This specification gives

$$\mu_{i}^{\mathrm{PH}}=E\left(y_{i}|v_{i}\right)=\left(1-\mu_{0,i}\right)\frac{\mu_{1,i}}{1-\exp[-\mu_{1,i}]}\;\text{and}\;\text{Var}\left(y_{i}|v_{i}\right)=\phi_{i}^{\mathrm{PH}}\mu_{i}^{\mathrm{PH}},$$

where $\phi_{i}^{\mathrm{PH}}=1+\left\{1-\left(1-\mu_{0,i}\right)/\left(1-\exp\left[-\mu_{1,i}\right]\right)\right\}\mu_{1,i}$. In hurdle models, $\mu_{0,i}>\exp\left[-\mu_{1,i}\right]$ implies $\phi_{i}^{PH}>1$, so that excessive zeros occur together with overdispersion.

As an alternative to the hurdle model, the ZIP and the zero‐inflated negative‐binomial (ZINB) models are often considered. Consider the ZINB model:

(8) $$\left.\begin{array}{ccc} P(y_{i}) & =p_{i}+\left(1-p_{i}\right)\exp\left[-\lambda_{i}\right], & y_{i}=0\\ P(y_{i}) & =\left(1-p_{i}\right)\frac{\exp\left[-\lambda_{i}\right]\lambda_{i}^{y_{i}}}{y_{i}!}, & y_{i}>0\\ \log\text{it}(p_{i}) & =Z_{i}\gamma & \\ \log(\lambda_{i}) & =X_{i}\beta +w_{i} & \end{array}\right\},$$

where covariates *Z*_*i*_ and *X*_*i*_ are the same as in the hurdle model and exp[*w*_*i*_] follows the independent gamma distribution with *E*(exp[*w*_*i*_]) = 1. When all *v*_*i*_ = 0, it becomes the ZIP model. Since

$$\mu_{i}^{\mathrm{ZIP}}=E\left(y_{i}|v_{i}\right)=p_{i}\lambda_{i}\;\text{and}\;\text{Var}\left(y_{i}|v_{i}\right)=\phi_{i}^{\mathrm{ZIP}}\mu_{i}^{\mathrm{ZIP}},$$

where $\mu_{i}^{\mathrm{ZIP}}=1+\left(1-p_{i}\right)p_{i}\lambda_{i}\geq 1$, this implies that ZIP and ZINB models only allow overdispersions.

### 2.4 Overdispersion due to random effects leads to higher probability of zero counts

So far, we have discussed the usefulness and limitations of ZIP and hurdle models for zero‐inflated data. Now, it remains to explain how a spatial HGLM can handle excessive zero counts. We explain this issue in two steps. First, we show that overdispersion due to random effects leads to zero inflation (see Theorem 1). Then, we explain how spatial correlation can lead to even higher proportions of zeros in observed data compared with independent observations.

Theorem 1 If *U*_*i*_, (*i* = 1, 2, …, *n*) is iid Poisson‐distributed with $E\left(U_{i}\right)=\exp\left[\eta_{i}\right]$, *V*_*i*_|*u*_*i*_ is also iid Poisson with $E\left(V_{i}|u_{i}\right)=\exp\left[\delta_{i}+u_{i}\right]$, *u*_*i *_~ *N*(0, σ^2^), and η_*i*_ = δ_*i*_ + (σ^2^/2) so that $E\left(U_{i}\right)=E\left(V_{i}\right)$ but *V*_*i*_ is overdispersed, for σ^2^ > 0 then, $\mathrm{Pr}\left(V_{i}=0\right)>\mathrm{Pr}\left(U_{i}=0\right)$.

Proof of Theorem 1 From the definition of marginal probability, we have

(9) $$\begin{array}{cc} & \mathrm{Pr}\left(V_{i}=0\right)=E\left(\mathrm{Pr}(V_{i}=0|u_{i})\right)=E\left(\exp[-\exp[\delta_{i}+u_{i}]]\right)\\ & =E\left(\sum_{k=0}^{\infty }\frac{1}{k!}{\left(-\exp[\delta_{i}+u_{i}]\right)}^{k}\right)=\sum_{k=0}^{\infty }\frac{1}{k!}{\left(-\exp[\delta_{i}]\right)}^{k}E\left(\exp{\left[u_{i}\right]}^{k}\right)\\ & \Rightarrow \mathrm{Pr}\left(V_{i}=0\right)=\sum_{k=0}^{\infty }\frac{1}{k!}{\left(-\exp[\delta_{i}]\right)}^{k}\exp\left(\frac{1}{2}\sigma^{2}k^{2}\right). \end{array}$$

The *k*th term of the summation on the right hand side of equation (9) is

(10) $$t_{k}=\frac{1}{k!}{\left(-\exp[\delta_{i}]\right)}^{k}\exp\left(\frac{1}{2}\sigma^{2}k^{2}\right).$$

When *k* is even, or 0, then $t_{k}\geq \frac{1}{k!}{\left(-\exp\left[\delta_{i}\right]\right)}^{k}$ because $\exp\left(\frac{1}{2}\sigma^{2}k^{2}\right)$ ≥ 1 for σ2k2 ≥ 0. Again, when *k* is odd, $t_{k}<\frac{1}{k!}{\left(-\exp\left[\delta_{i}\right]\right)}^{k}$. But,

(11) $$\exp\left[-\exp[\delta_{i}]\right]=\sum_{k=0}^{\infty }\frac{1}{k!}{\left(-\exp[\delta_{i}]\right)}^{k}.$$

Comparing equations (9) and (11), we see that all the positive terms (for *k *=* *0, 2, 4, …) in the summation series on the right hand side of equation (9) are greater than (or equal to when *k* = 0) the corresponding terms in equation (11) and all the negative terms (for *k *=* *1, 3, …) in equation (9) are smaller than the corresponding terms in equation (11). Therefore, we have

$$\sum_{k=0}^{\infty }\frac{1}{k!}{\left(-\exp[\delta_{i}]\right)}^{k}\exp\left(\frac{1}{2}\sigma^{2}k^{2}\right)>\exp\left[-\exp[\delta_{i}]\right]>\exp\left[-\exp\left[\delta_{i}+\frac{\sigma^{2}}{2}\right]\right].$$

But, Pr(*U*_*i*_ = 0) = exp [−exp [η_*i*_]] = $\exp[-\exp[\delta_{i}+\frac{\sigma^{2}}{2}]].$ Therefore, $\exp[-\exp[\delta_{i}+\frac{\sigma^{2}}{2}]].$.

From Theorem 1, we see that overdispersion due to random effects leads to a higher probability of zero counts, in other words zero inflation, compared with an ordinary Poisson GLM. To illustrate how spatial correlation can introduce an even higher proportion of zeros, let us consider two observations, *Y*_1_ and *Y*_2_ om a binary variable (0 represents zero counts, 1 represents nonzero), then it is straightforward to show that (proof is omitted) $\mathrm{Pr}\left(Y_{1}=0\;\&\;Y_{2}=0|\text{Cor}\left(Y_{1},\;Y_{2}\right)>0\right)>\mathrm{Pr}\left(Y_{1}=0\;\&\;Y_{2}=0|Y_{1}⊥Y_{2}\right)$. In other words, due to spatial correlation, co‐occurrence of zero counts can give higher proportions of zeros in some samples than expected in the case of independent observations.

A reviewer pointed out that an additional covariate could also explain excessive zeros in the data. This is true, but herein we assume that the relationships between the mean of the response and the covariates are correctly specified, and only the assumptions about the distribution should be questioned. In this respect we have in mind that in real data analysis we do not have many options about the covariates and link functions, only thing we can do is to try to improve the fit of our model by adopting different families of distributions for the response variable.

### 2.5 Analysis of reindeer pellet‐group counts

**Figure 1:**
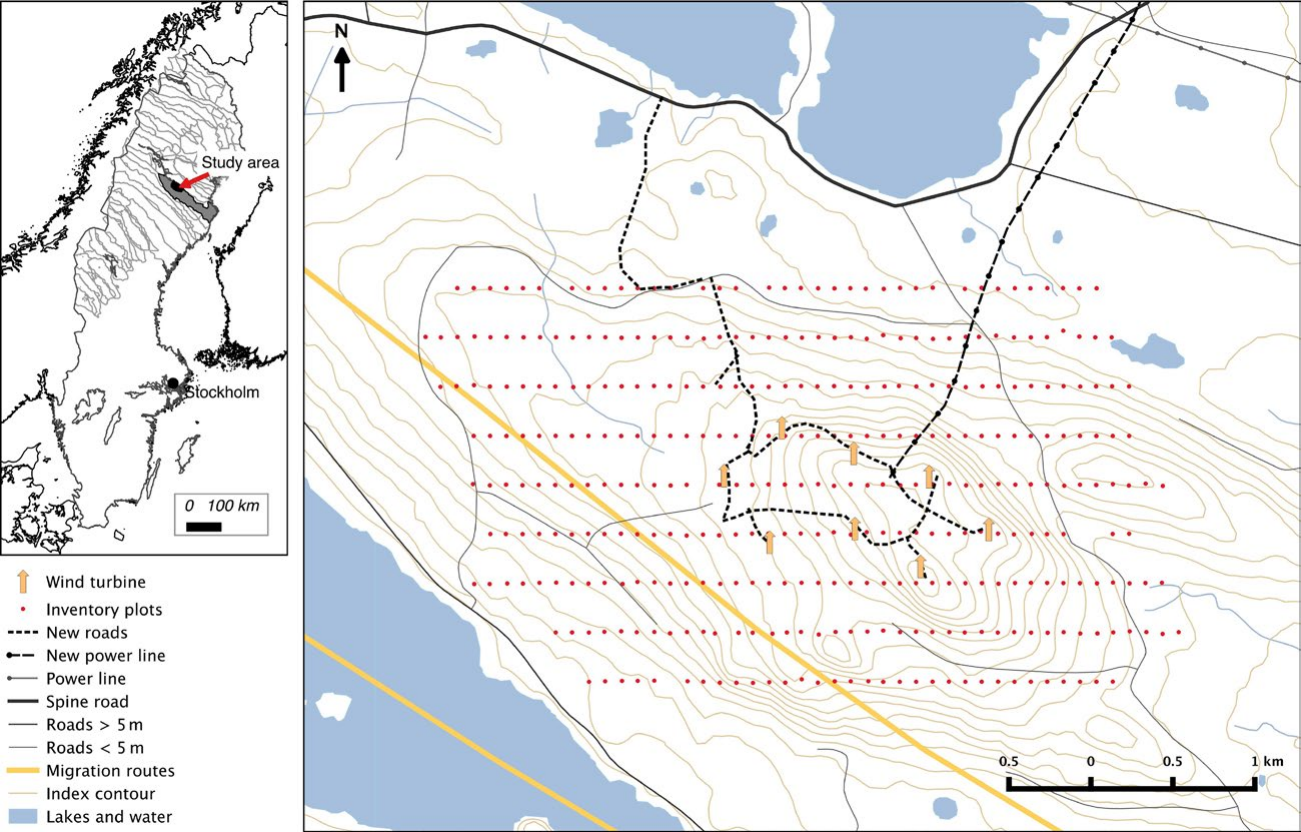
Map of the study area and the inventory plots for the pellet‐group count conducted on Storliden Mountain in the Malå reindeer herding community. Location of the study area in Sweden is shown in the smaller map. © Lantmäteriet i2014/764

We analyze a real data set pertaining to reindeer fecal pellet‐group counts. The data were obtained from a survey conducted on Storliden Mountain (504 m MLS; 65°13′N, 18°53′E) in northern Sweden (see Fig. 1). The size of the study area was 25 km^2^, and eight windmills were built in the center of the area in 2011. The survey was conducted between 3 and 8 June in 2009 and 28 May and 1 June in 2010. Reindeer graze freely in this area from May to October except during a short period in early July when they are gathered for the marking of the calves. The survey was conducted using a point transect design (Buckland et al., 2001) and was part of a larger inventory of reindeer pellet groups over an area of 250 km^2^. The distance between each transect was 300 m and the distance between each plot (red dots, in Fig. 1) on each transect was 100 m. Each plot had a size of 15 m^2^ (radius = 2.18 m). The coordinates of the plots were recorded, and the center of each plot was marked with an orange wooden stick.

The pellet groups were counted using the fecal standing crop (FSC) technique in 2009 and fecal accumulation rate (FAR) in 2010 (see definitions in Skarin, 2008). A pellet group was counted for a certain plot if the center of the group was found inside the plot. Because an animal might move as it defecates, the pellets can spread over a large area. Therefore, a pellet group was defined by a cluster of 20 or more pellets.

Preference for habitat use by reindeer, based on the pellet‐group counts, was modeled for each inventory. From the initial analysis (not reported), it was noted that 73.67% of the plots had zero counts in 2009 and 83.62% had zero counts in 2010. This indicates (possible) inappropriateness of standard count data models, for example, the Poisson GLM. Although our FSC inventory probably had a higher abundance in dry vegetation types due to the slower decay rate of the pellets (Skarin, 2008), we did not take this into account here, as the main purpose of this research was to find a method to treat the large amount of zeros in the data.

## 3 Results

### 3.1 Spatial modeling of reindeer pellet‐group counts

In order to deal with the issues of spatial correlation and excessive counts, with the pellet group data, we apply three different models for overdispersion (see Table 1). The spatial covariance structure for the normal random effects is either $\Sigma^{-1}=\frac{1}{\tau }\left(I-\rho D\right)$ (i.e., CAR, which includes Poisson‐normal HGLM as a special case for ρ = 0) or $\Sigma^{-1}=\sigma^{2}{\left({\left(I-\rho D\right)}^{T}\left(I-\rho D\right)\right)}^{-1}$. We also fit a Poisson GLM, as would be done in regression kriging (Bivand, Pebesma, & Gomez‐Rubio, 2008). A detailed list of the fitted models is presented in Table 1.

**Table 1:** Specifications of the fitted models

Model	Description	Mean ($\eta_{i}=\log\left(\lambda_{i}\right)$)	Variance function ($V(\lambda_{i})$)	Random effects and their distributions
I	Poisson GLM	*X*_*i*_β	λ_*i*_	No
II	Poisson HGLM	*X*_*i*_β + *v*_*i*_	λ_*i*_	$v^{T}=\left(v_{1},v_{2},\ldots ,v_{n}\right)$, *v*_*i*_ ~ *N*(0, τ)
III	Poisson HGLM with CAR	Model II	λ_*i*_	v∼N(0,Σ), $\Sigma^{-1}=\left(1/\tau \right)\left(I-\rho D\right)$
IV	Negative‐binomial HGLM with CAR	*X*_*i*_β + *v*_*i*_ + *w*_*i*_	λ_*i*_	$\exp\left[w_{i}\right]∼\text{Gamma}\left(\alpha ,1/\alpha \right)$, **v** is as in Model III
V	Quasi‐Poisson HGLM with CAR	Model II	ϕλ_*i*_	Same as Model III

As GLMs are uniquely specified by the mean (in our case, *λ*_*i*_) and the variance function, *V*(*λ*_*i*_), we use only these parameters and functions to specify the models. All the spatial models presented in Table 1 can be fitted using the HGLM algorithm (an R implementation is available in the supplementary material).

For the 2009 data, we started with a large (full) model containing 13 covariates: the (log‐) distance from the power grid, slope of the location, a ruggedness index, elevation, forest age structure, dummies (1/0) for clear‐cuts, young forest, coniferous forest, broad‐leaved forest, flat area, southeast slope, northwest slope, and northeast slope. To avoid possible multicollinearity, we did not include (log) distance to nearby big infrastructure (which had a correlation of 0.8 with log‐distance to power grid, while the correlation between any other pairs of variables did not exceed 0.6, in absolute value) in the model. For the 2010 data, we excluded the dummy variable for broad‐leaved forest from the full model. In the 2010 data, broad‐leaved forests had only pellet‐group counts of zero; this indicates that special care is necessary to tackle the exploding tendency of the ML estimate, if it exists, for the relevant parameter (Feinberg & Rinaldo, 2007). However, we found that the MLEs of the other parameters remained similar after the broad‐leaved forest was dropped from the model.

Table 2 reveals that the quasi‐Poisson‐normal HGLM with ϕ < 1 and the CAR (QCAR) specification (Model V) fits the data best, as it has the lowest cAIC. The models with the SAR covariance structure are not able to produce a better fit than the QCAR model. We do not, therefore, report those results in this paper.

**Table 2:** cAIC with full set of covariates

Model	cAIC^a^	
	2009 data	2010 data
Model I	628.71	432.13
Model II	587.93	402.84
Model III	586.55	406.15
Model IV	542.10	326.18
Model V	528.87	206.77

a cAIC iss the AIC for model I.

Starting with the best‐fitted full model, we gradually delete covariates one at a time from the model on the basis of the absolute *t*‐value (covariate with the lowest absolute *t*‐value deleted first as commonly suggested in the literature, see, e.g., McCullagh & Nelder, 1989; Ch. 3.9) until we obtain the final model, having all the fixed‐effect parameters significant at the 5% level (both in the Wald and likelihood‐ratio tests). The estimated parameters and their standard errors for the final models for the 2009 and 2010 data are presented in Table 3.

**Table 3:** Estimated model parameters and fit statistics for Model I and Model V (final)

Parameters	For 2009 FSC counts		For 2010 FAR counts	
	Model I	Model V	Model I	Model V
Intercept	−18.916 (3.782)	−18.171 (4.491)	−12.21 (4.562)	−10.979 (5.163)
Northwest slopes	−0.489 (0.304)	−0.656 (0.364)		
Southeast slope			0.696 (0.316)	0.988 (0.410)
Elevation	0.007 (0.002)	0.007 (0.003)	−0.005 (0.003)	−0.005 (0.004)
Distance to power lines	1.897 (0.426)	1.728 (0.519)	1.569 (0.499)	1.33 (0.595)
Clear‐cuts			0.607 (0.346)	0.567 (0.500)
τ		1.324 (0.270)		2.327 (0.393)
ϕ		0.737 (0.082)		0.476 (0.036)
ρ		3.038 (0.750)		1.903 (4.416)
cAIC^a^	618.4	526.25	418.78	221.44

Values in parentheses represent standard error.

a cAIC is AIC for GLM.

From the results (Table 3), we see that distance from power grid was the most influential factor (both in 2009 and 2010), and it was also statistically significant (*p*‐value < .001 for both GLM and QCAR). Its positive coefficient estimate reveals that the pellet‐group counts were higher at locations farther away from power lines.

**Figure 2:**
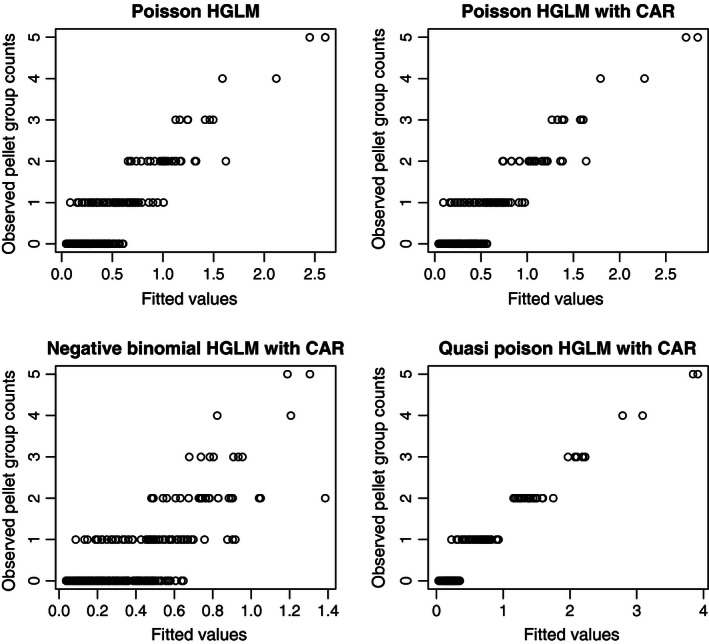
Plots of observed versus fitted values for different models (with the full set of covariates) for the 2009 FSC count

A plot of the observed responses against the fitted values (for the 2009 FSC count) is given in Fig. 2. The same plots for the 2010 FAR count data reveal the same overall pattern, so these plots are not shown. From Fig. 2, we see that the fit of the model gradually improves as the spatial dependence structures we incorporate become more reasonable. This indicates the advantage of joint modeling of the mean and the covariance. By comparing the plots for the four models in Fig. 2, we see that QCAR (lower right in Fig. 2; which is also the best fit model in terms of cAIC, see Table 2) not only improves the mean prediction but also reduces the predicted mean square error (PMSE; see Table 4).

**Table 4:** Average PMSE of various models, based on 100 random test data sets

Model	PMSE for 2009 data	PMSE for 2010 data
Model I	0.665	0.351
Model II	0.663	0.349
Model III	0.272	0.201
Model IV	0.142	0.154
Model V	0.123	0.115
Hurdle	0.606	0.380^a^
ZIP	0.620	0.355
ZINB	0.621	0.355

a Calculated after ignoring four abnormal (>1,000) PMSEs.

Comparing the four plots of the observed counts with the in‐sample fitted values in Fig. 2, we see that the simple Poisson‐normal HGLM (upper left plot in Fig. 2) was not able to model excessive zero counts, adequately. Harrison (2014) also showed, in a simulation study, that this Possion‐normal HGLM failed to reduce bias in zero‐inflated data. By modeling spatial correlation (Poisson‐normal HGLM with CAR), we get a better fit compared with the Poisson‐normal HGLM with independent random effects. Figure 2 also shows that the excessive overdispersion that results from using a negative‐binomial‐normal HGLM with CAR does not improve the prediction. Finally, QCAR gives the best fit. The same findings hold for both the 2009 and 2010 data. Here, $\hat{\phi }=0.737<1$ for 2009 and $\hat{\phi }=0.476<1$ for 2010.

### 3.2 Comparison with the hurdle model

The hurdle model is frequently used for analyzing count response with excess zeros. Therefore, we also analyze the reindeer pellet‐group counts using such a model (eq. (7)). We assume $v_{k}∼N\left(0,\Sigma_{k}\right)$ where Σ_1_ has a CAR specification as in Model III (see Table 1). Because no R package module is able to fit Model (7) with CAR random effects in a non‐Bayesian manner, we developed our own R codes to carry out the model computation using a hierarchical likelihood (h‐likelihood) approach (a brief description of the algorithm, and the R program are available in the supplementary material).

**Figure 3:**
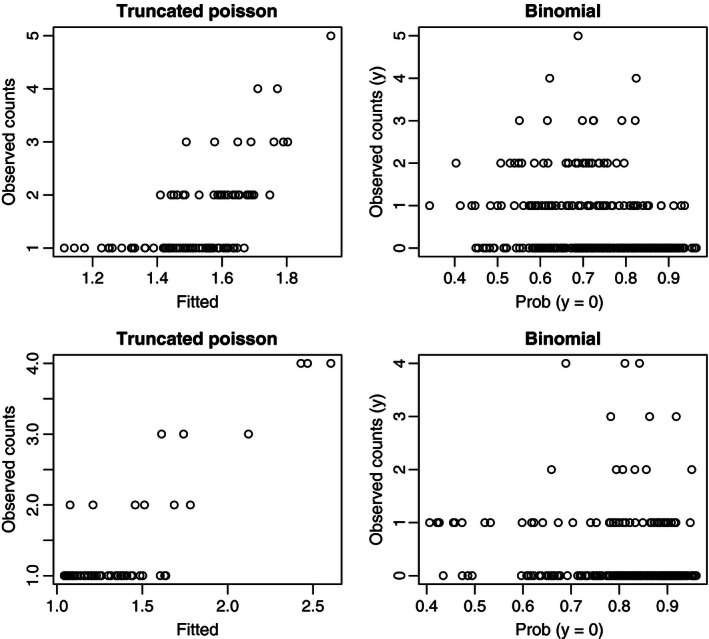
Plots of observed versus fitted values for the binomial and truncated Poisson parts of the hurdle models for the FSC (Year 2009, top panel) and FAR (Year 2010, bottom panel) counts

For the binary part (*μ*_0,*i*_ in eq. (7)), we use four covariates (northwest slope, southeast slope, elevation, and log‐distance to power grid) for the 2009 data and six covariates (southeast slope, Young forest, clear‐cuts, forest age structure, elevation, and log‐distance to power grid) for the 2010 data. These variables were selected on the basis of separate binomial models for Pr(Count > 0) and because they have the lowest cAIC values. For the truncated Poisson model in equation (7), we use the same set of covariates as in Table 3. Because the truncated Poisson part of the hurdle model does not provide a direct estimate of the random effects for the locations with observed 0 counts, a direct computation of the fitted values for the hurdle model is not straightforward (a kriging approach as presented in Section Conflict of Interest could be an option though). Therefore, we report the fit of the binomial and the truncated Poisson part of the hurdle models separately in Fig. 3. The top panel of Fig. 3 shows the fit of the binomial (left) and truncated Poisson (right) parts of the hurdle model for the 2009 data. The bottom panel of Fig. 3 shows the same for the 2010 data.

Comparing Figs 2 and 3, we see that the HGLM with QCAR (Model V) provided a better fit than the hurdle model. Although the truncated Poisson part of the hurdle model did a reasonably good job, the failure in the binary part downgraded the overall prediction. We tried, using all the available variables, to improve the performance of the binary part of the model, but we failed. We could not fit a spatial correlation in the binary part because standard R packages, for example, lme4 (Bates, Maechler, Bolker, & Walker, 2015) and hglm (Alam, Ronnegard, & Shen, 2015), cannot fit a binary GLMM with one observation per subject. If repeated observations from each plot were available, we could try to apply bivariate random effects (for binary part and Poisson part), as used in Neelon et al. (2013), but with the current data set we cannot improve on our current approach.

### 3.3 Comparison with a zero‐inflated Poisson model

**Figure 4:**
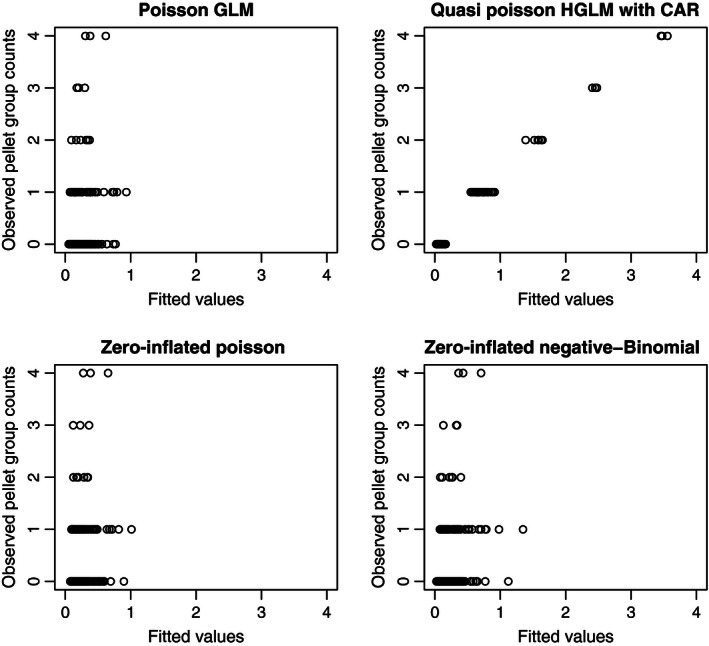
Plots of fitted versus observed values for Poisson GLM, QCAR, ZIP, and ZINB for the 2010 FAR counts

Figure 4 shows that the HGLM with QCAR (Model V) provided a better fit than Poisson GLM, ZIP, and ZINB models. The hurdle model allows overdispersion only for zero‐deflation cases. However, this data set exhibits underdispersion but with excessive zeros. This means that ZIP, ZINP, and hurdle models are not appropriate to apply to this data set. The quasi‐Poisson HGLM allows for underdispersion (ϕ < 1) with excessive zeros.

### 3.4 Prediction by the models

To evaluate the performance of predictions from various models, the whole data set is divided randomly into two parts: 70% as the data form training set and the remaining 30% form the test set; this division is repeated for 100 times. After fitting models I–V to each training set, the PMSE is computed for the rest of the data (the test set) using the following formula

(12) $$\text{PMSE}=\frac{1}{n_{\mathrm{test}}}\sum_{i=1}^{n_{\mathrm{test}}}{\left(y_{i}-{\hat{\lambda }}_{i}\right)}^{2},$$

where *n*_test_ is the sample size of the test set, *y*_*i*_ is the response of the test set and $\hat{\lambda }$ is the estimator of λ_*i*_ using the training set. For models I–II without a spatial correlation, $\lambda_{i}=\exp\left[X_{i}\hat{\beta }\right]$ where $\hat{\beta }$ is the estimated β from the training set. For models III–V with a spatial correlation, $\lambda_{i}=\exp\left[X_{i}\hat{\beta }+{\hat{v}}_{i}\right]$ where ${\hat{v}}_{i}$ is the predicted value of *v*_*i*_. We compute ${\hat{v}}_{i}=\hat{\text{cov}}\left(v_{i},v_{i}^{\mathrm{Train}}\right)\hat{\text{cov}}{\left(v_{i}^{\mathrm{Train}}\right)}^{-1}{\hat{v}}_{i}^{\mathrm{Train}}$ where ${\hat{v}}_{i}^{\mathrm{Train}}$ is the random location effect of the training set. Here, $\hat{\text{cov}}{\left(v_{i}^{\mathrm{Train}}\right)}^{-1}$ and ${\hat{v}}_{i}^{\mathrm{Train}}$ are estimated using the training set, and $\hat{\text{cov}}\left(v_{i},\;v_{i}^{\mathrm{Train}}\right)$ is $\text{cov}(v_{i},\;v_{i}^{\mathrm{Train}})$ after replacing the parameters involved with their estimates from the training set.

Table 4 shows the average PMSE of 100 random selections of the training and test sets. HGLM with QCAR (Model V) with underdispersion and spatial correlation is, overall, the best‐fitting model giving the lowest cAIC (also known as DIC, see Lee et al., 2006, Ch. 6.5; see also the supplementary materials) and PMSE, together. Thus, the model with underdispersion gives better predictions than overdispersed models with zero inflation.

## 4 Discussion

In this paper, we introduce a quasi‐Poisson HGLM with a spatial correlation to fit reindeer pellet‐group counts, and we show that a Poisson GLM, by ignoring spatial correlation, can lead to a poor model fit. Such a simplified model produces poor‐quality residuals (due to the lack of fit), which lead to incorrect conclusions being drawn about the spatial correlation. Consequently, the regression kriging prediction based upon those residuals, which is often suggested in the literature (see, e.g., Bivand et al., 2008; Cressie, 1993; Gribko, Hohn, & Ford, 1999), may result in poor spatial prediction.

From the results of the fitted quasi‐Poisson HGLM (see Table 3), we conclude that several environmental variables, for example, slope, elevation, and vegetation type at the location, as well as human development activities, for example, power lines, are significant factors explaining reindeer habitat preference.

In the literature, hurdle and ZIP models are widely used for analyzing count responses with excessive zeros. However, hurdle and ZIP models do not allow for underdispersion with excessive zeros. In practice, such data sets often exist, for example, the incidence rate of hospitalization (Tin, 2008), and accident rates when accidents are very rare events (Oh et al., 2006), where excess zero counts appear along with underdispersion.

For a real data set pertaining to pellet‐group counts, we fit models with zero inflation, ZIP and ZINB. However, they give poor predictions (Table 4) and have poor fitted values (Fig. 4). Spatial‐correlation‐only models (III and IV) improve both prediction and fitted values. However, hurdle, a model with both zero inflation and spatial correlation, is worse than a spatial‐correlation‐only model. We show that Poisson HGLM with spatial correlation and underdispersion, namely Model V, provides the best predictions and fitted values.

With the reindeer pellet‐group counts, a quasi‐Poisson HGLM with CAR allows for underdispersion (ϕ < 1) with excessive zeros for both 2009 and 2010 data. Thus, quasi‐Poisson HGLM with CAR random effects provides a better fit with the data than the hurdle model with similar linear covariates and correlation structures. Our results, however, do not imply that Poisson HGLM can be safely used to analyze data that are generated by a true zero‐inflated Poisson or a hurdle model (see contrasting example in Zuur et al., 2012). If the underlying subject matter theory leads to a ZIP or a hurdle model, then that model should be applied. However, the results show that we cannot reject a HGLM in favor of a ZIP or a hurdle model only because the data contain a high proportion of zeros; overdispersion, high correlation, and a covariate may well be able to explain the excessive zeros.

It would be interesting in future work to extend hurdle models to allow for underdispersion with excessive zeros by adopting some sort of weighted Poisson distribution (Ridout & Besbeas, 2004; and references cited therein), generalized Poisson, or gamma distribution (Oh et al., 2006) for the positive response part. However, computation of those models, especially when there is spatial dependence, and the interpretation of the model parameters would be challenging tasks. Spatial hurdle models are commonly fitted using Bayesian MCMC techniques (Zuur et al., 2012), which are computationally too intensive. If there is no special reason for using a Bayesian approach (such as priors originating from a theoretical justification), one can use an HGLM model computed using the h‐likelihood method that provides a deterministic algorithm (R code is provided in the supplementary material with this paper) and is faster than conventional MCMC methods.

## Conflict of Interest

None declared.

## Data Accessibility

The reindeer pellet‐group survey data set used in this article is available in the supplementary material.
