# Supplementary material for: Spatial modeling of data with excessive zeros applied to reindeer pellet‐group counts
Source: Ecol Evol. 2016 Sep 12;6(19):7047–56. doi: 10.1002/ece3.2449 (PMC5513232; doi:10.1002/ece3.2449)
Supplement: Supplementary file 9 [file ECE3-6-7047-s009.pdf]

Supplementary material: Spatial modelling of data with excessive zeros applied to reindeer pellet-group counts

Youngjo Lee [1], Md. Moudud Alam [2,*], Maengseok Noh [3], Lars Rönnegård [2] and Anna Skarin [4]

Affiliations

1. Department of Statistics, Seoul National University, Seoul, Republic of Korea
2. Dalarna University, School of Technology and Business Studies, Falun, Sweden
3. Department of Statistics, Pukyong National University, Busan, Republic of Korea
4. Department of Animal Nutrition and Management, Swedish University of Agricultural Sciences, Uppsala, Sweden

Corresponding author: Md. Moudud Alam, Dalarna University, School of Technology and Business Studies, SE-791 88 Falun, Sweden

Phone: +46-725468854

Email: maa@du.se

Estimation procedure for HGLMs

Consider the models the following model, in matrix notation

$$g(\lambda) = X\beta + Zv \tag{1}$$

where $E(\mathbf{z}|\mathbf{v}) = \lambda$ with $\mathbf{z}^T = (z_1, z_2, \ldots, z_n)$ and $\lambda^T = (\lambda_1, \lambda_2, \ldots, \lambda_n)$, X is the model matrix for the fixed effects β and Z is the model matrix associated with $\mathbf{v}$. For inferences about HGLMs we use the h-likelihood presented by Lee & Nelder (1996) as

$$h = \sum_{i=1}^{n} \log f(z_i|v_i) + \log f(\mathbf{v}). \tag{2}$$

For estimation of fixed parameter β, we use the first-order Laplace approximation $p_v(h)$ defined below to the marginal likelihood $m = \log \int \exp(h) dv$

$$p_v(h) = \left(h - \frac{1}{2} \log\{|\frac{\partial^2 h}{\partial v \partial v^T}|/(2\pi)\} \right)_{v=\hat{v}}, \tag{3}$$

where $\hat{v}$ is the solution of $\partial h/\partial v = 0$. For estimation of the dispersion parameters θ (consisting of τ, ρ, ϕ, and α), we maximize the adjusted profile h-likelihood

$$p_{\beta,v}(h) = \left(h - \frac{1}{2} \log\{|\frac{\partial^2 h}{\partial (v,\beta) (\partial v, \beta)^T}|/(2\pi)\} \right)_{v=\hat{v},\beta=\hat{\beta}} \tag{4}$$

which gives an extension of restricted maximum (log-)likelihood (Lee *et al.*, 2006). The above estimation algorithm can be implemented in a two-step procedure (Lee *et al.*, 2006). In the first step, an iterative weighted least square (IWLS) is implemented for estimating v, β by maximizing h and $p_v(h)$, respectively given dispersion parameters θ. In the second step, $p_{\beta,v}(h)$ (4) is maximized to estimate θ. The procedure iterates between these two steps until

convergence.

Because we consider spatial data where there are as many random effects as the number of observations, a simple penalized quasi-likelihood (Breslow & Clayton, 1993) introduces serious bias in the parameter estimates (see further discussion in Lee & Lee, 2012). Thus, in this paper, we use the extended Fisher's scoring algorithm described in Lee & Lee (2012) , with necessary modification to handle a quasi-Poisson model, for fitting generally structured spatial models.

Molas & Lesaffre (2010) showed that h-likelihood approach enables us to fit the hurdle mixed model by using the same iterative algorithm as above, given that $\mathbf{v}_0 \perp \mathbf{v}_1$ and $v_{k,i} \perp v_{k,j}$, $\forall i \neq j$ and k. For $\mathbf{v}_k$ distributed as Gaussian CAR (or SAR) with variance-covariance parameters τ_k and ρ_k then by using an eigenvalue decomposition of $\mathbf{D}$ matrix we can reformulate the linear predictors in the hurdle model as

$$g_k(\mu_i) = \mathbf{X}_i\beta_k + \tilde{\mathbf{Z}}_i\tilde{\mathbf{v}}_k \tag{5}$$

where $\tilde{\mathbf{Z}}_i = \mathbf{Z}\mathbf{\Gamma}$ with $\mathbf{\Gamma}$ being a matrix whose columns are the eigenvectors of $\mathbf{D}$ and $\tilde{\mathbf{v}}_k = \mathbf{V}^T\mathbf{v}_k$. This representation reduces each element in $\tilde{\mathbf{u}}_k = \{u_{\tilde{k},i}\}$ to independently distributed as $N(0, \phi_{k,i})$ where $\nu(\phi_{k,i}) = \theta_{0,k} + \theta_{1,k}\lambda_i$, $\nu(x) = 1/x$ is an inverse link function for CAR ($= 1/\sqrt(x)$ for SAR), $\theta_{0,k} = \frac{1}{\tau_k}$ ($= \frac{1}{\sqrt{\tau_k}}$ for SAR) and $\theta_{1,k} = \frac{-\rho_k}{\tau_k}$ ($= \frac{-\rho_k}{\sqrt{\tau_k}}$ for SAR). This representation with independent $\widetilde{v}_{k,i}$ enables us to use the same IWLS algorithm as Molas & Lesaffre (2010) with minor modification to handle the heteroscedastic random effects, $\widetilde{v}_{k,i}$.

Therefore, h-likelihood approach enables us to fit both the spatial Poisson and the spatial hurdle model with almost identical computational procedure. This computational procedure is very fast, compared to the Bayesian Markov Chain Monte Carlo based procedures which are often use in analyzing zero inflated count response (see e.g., Neelon *et al.*, 2013; Agarwal *et al.*, 2002). An

R (R Development Core Team, 2015) implementation of the above algorithm is provided as supplementary material (we also plan to make it available on CRAN). A slightly different implementation of the CAR model (for HGLMs but not for hurdle) is provided in **R** package **hglm-v2.1** Alam *et al.* (2015). Spatial HGLMs can also be fitted by using **spaMM** package Rousset & Ferdy (2014) in **R**. However, **spaMM** does not allow fitting quasi models, therefore, we could not use it for data analysis. In the following subsection, we discuss the model selection techniques used with HGLMs.

Model selection for HGLMs

For model selection, Lee *et al.* (2006) proposed various statistics based upon h-likelihood, e.g., for estimation and model selection of variance and covariance models, the adjusted profile h-likelihood $p_{\beta,v}\left(h\right)$ defined in (4) is suggested; for the estimation of fixed effects, β, $p_v(h)$ is suggested. For nested models, differences in $-2p_v\left(h\right)$ can be treated the same way as is done for the classical likelihood ratio test. For non-nested models we can use information criteria, such as the conditional Akaike's information criteria (cAIC), based upon the first component of h-likelihood, which is given as

$$cAIC = d + 2p_d \tag{6}$$

where $d = -2\left(l\left(\hat{\lambda};\mathbf{z}|\mathbf{v}\right) - l\left(\mathbf{z};\mathbf{z}|\mathbf{v}\right)\right)$, $l\left(\hat{\lambda};\mathbf{z}|\mathbf{v}\right) = \log f\left(\mathbf{z}|\mathbf{v};\hat{\lambda}\right)$, and $n - p_d$ is the degrees of freedom for the deviance d Lee *et al.* (2006), with $p_d = trace\left(H^{-1}H^*\right)$; $H = -2\frac{\partial h^2}{\partial(\beta,\mathbf{v})\partial(\beta,\mathbf{v})^T}$ and

$$H^* = \begin{bmatrix} X^TWX & X^TWZ \\ Z^TWX & Z^TWZ \end{bmatrix}$$

where X and Z are as in equation (1) and W is the diagonal GLM weight matrix (McCullagh & Nelder, 1989), whose i^{th} diagonal element is given by $W_{i,i} = \left(\frac{\partial^2 \eta}{\partial \lambda_i^2}\right)^2 V^{-1}(\lambda_i)$ where V is the GLM variance function (McCullagh & Nelder, 1989). Spiegelhalter *et al.* (2002) refer to the cAIC as the deviance information criterion in connection to selection of Bayesian models, whereas Donohue *et al.* (2011) use it for frequentist model selection.

References

Agarwal, D. K., Gelfand, A. E. & Citron-Pousty, S. (2002). Zero-inflated models with application to spatial count data. *Environmental and Ecological Statistics*, 9, 341–355.

Alam, M. M., Rönnegård, L. & Shen, X. (2015). Fitting conditional and simultaneous autoregressive spatial models in hglm. *The R Journal*, 7(2), 5–18.

Breslow, N. E. & Clayton, D. G. (1993). Approximate inference in generalized linear mixed models. *Journal of the American Statistical Association*, 88, 9–25.

Donohue, M. C., Overholser R., Xu, R. & Vaida, F. (2011). Conditional akaike information under generalized linear and proportional hazards mixed models. *Biometrika*, 98, 685–700.

Lee, W. & Lee, Y. (2012). Modifications of reml algorithm for hglms. *Statistics and Computing*, 22, 959–966.

Lee, Y. & Nelder, J. A. (1996). Hierarchical generalized linear models (with discussion). *Journal of the Royal Statististical Society, Series B*, 58, 619–656.

Lee, Y., Nelder, J. A. & Pawitan, Y. (2006). *Generalized Linear Models with*

Random Effects: Unified Analysis via H-likelihood. Chapman & Hall/CRC, Boca Raton.

McCullagh, P. & Nelder, J. A. (1989). *Generalized Linear Models.* Chapman & Hall, London.

Molas, M. & Lesaffre, E. (2010). Hurdle models for multilevel zero-inflated data via h-likelihood. *Statistics in Medicine*, 29, 3294–3310.

Neelon, B., Ghosh, P. & Loebs, F. P. (2013). A spatial poisson hurdle model for exploring geographic variation in emergency department visits. *Journal of the Royal Statistical Society, Series A*, 176, 389–413.

R Development Core Team (2015). *R: A language and environment for statistical computing.* R Foundation for Statistical Computing, Vienna, Austria, http://www.R-project.org/.

Rousset, F. & Ferdy, J. (2014). Testing environmental and genetic effects in the presence of spatial autocorrelation. *Ecography*, 37, 781–790.

Spiegelhalter, D. J., Best, N. G., Carlin, B. P. & van der Linde, A. (2002). Bayesian measure of model complexity and fit (with discussion). *Journal of the Royal Statistical Society, Series B*, 64, 583–640.
